# In the trail of the crime scene dog: Processing of human DNA from outdoor samples

**DOI:** 10.1016/j.fsisyn.2026.100664

**Published:** 2026-02-12

**Authors:** Christina Forsberg, Ronny Hedell, Ricky Ansell, Johannes Hedman

**Affiliations:** a National Forensic Centre, Swedish Police Authority, Linköping, Sweden; b Division of Biotechnology and Applied Microbiology, Department of Process and Life Science Engineering, Lund University, Lund, Sweden; c Department of Physics, Chemistry & Biology, Linköping University, Linköping, Sweden

**Keywords:** DNA extraction, Forensic DNA analysis, Search dog, Semen-detection dog, Soil

## Abstract

The growing use of crime scene dogs to detect human body fluids has led to an increased number of outdoor samples being submitted to forensic DNA laboratories. The analysis of DNA in outdoor samples such as soil presents significant challenges with present protocols due to the combination of relatively large sample volumes and co-extraction of humic substances. To address this, we have optimised a soil microbial DNA extraction protocol for the recovery of semen by incorporating enzymatic, chemical and physical lysis steps. This optimisation increased the DNA yield 43-fold. Using mock crime scene samples, the protocol provided high quality STR profiles from semen in 10 mL of different types of soil (mixed forest soil, sandy soil and park soil), organic matrix (dry leaves) and inorganic matrices (sand and gravel). The protocol also generated complete STR profiles for soil samples containing blood, saliva and cell-free DNA. However, all humic substances were not removed, causing inhibition in MPS analysis. The extracted DNA consisted of more than 95% double-stranded (ds) DNA with equivalent fragment sizes as DNA extracted using magnetic bead-based kits. The protocol is thus compatible with library preparation methods that demand dsDNA, e.g. in whole genome sequencing. The protocol has been successfully implemented in casework and used in serious crime investigations. Human STR profiles have been generated from crime scene items in 50% of the cases. In the majority of these (82%), no results were generated using swabs and traditional DNA extraction methods.

## 1 Introduction

Forensic DNA profiling is a highly effective tool in criminal investigations. Current methods enable the generation of complete short tandem repeat (STR) profiles from minute quantities of human biological material [1,2]. These profiles can be compared with individuals connected to the crime, as well as with profiles registered in national and international law enforcement DNA databases [3]. However, the successful generation of an STR profile is dependent on the ability of examiners to locate and collect biological traces at the crime scene. Outdoor crime scenes make this task especially challenging. These often involve larger search areas where evidence may be more difficult to detect and recover compared to indoor crime scenes. Moreover, DNA deposited outdoors is subject to accelerated degradation due to environmental factors such as humidity, microorganisms and ultraviolet (UV) radiation [[4], [5], [6]]. Additionally, outdoor sampling often results in the co-collection of environmental matrices, such as soil. These matrices contain high levels of polymerase chain reaction (PCR) inhibitors, further complicating DNA extraction and analysis [7,8].

In indoor environments, the search for biological stains such as semen is traditionally conducted using forensic light sources in combination with presumptive tests. Forensic light sources offer several advantages: they are rapid, non-destructive, and contact-free [9]. However, their effectiveness in outdoor settings is limited by the need for a dark environment. In addition, fluorescence from environmental matrices may lead to false positives and interfering substances may cause false negatives [10]. Due to practical constraints, presumptive testing is not feasible across large outdoor areas, creating a need for alternative screening methods [11].

In this context, trained crime scene dogs can serve as “intelligent samplers”, effectively narrowing down large areas to smaller spots of interest for further examination [12]. Canines possess 20 to 60 times more olfactory receptor cells than humans and are capable of detecting significantly smaller amounts of odours [[13], [14], [15]]. The human utilisation of the canine sense of smell can be traced back as far as 12,000 years for the purpose of hunting [16]. Today, the application of dogs' olfactory capabilities spans private, military and law enforcement sectors. Law enforcement use of search dogs includes detection of narcotics, weapons, explosives, accelerants, banknotes, human remains and human body fluids [9,[16], [17], [18], [19], [20], [21], [22], [23], [24], [25], [26]]. The effectiveness of crime scene dogs in detecting body fluids has been evaluated in several studies e.g. [[9], [11], [26]]. Notably, these dogs are capable of operating effectively in both full daylight and low-light conditions [13]. The increased use of crime scene search dogs has resulted in a corresponding rise in outdoor samples submitted to forensic DNA laboratories. In Sweden, this trend became particularly evident with the introduction of dogs trained for semen detection. The first such canine, named “Xena”, was active in the early 2000's [27]. In addition to semen-detection dogs, the Swedish Police Authority has trained dogs to detect blood and saliva [28].

Retrieved from an outdoor environment, (potential) body fluids deposited on a variety of different types of matrices may be relevant for forensic examination. These matrices include organic plant material such as moss, leaves, grass, and branches, inorganic material such as sand, gravel and stones as well as soil. Soil, in particular, has a drastic negative impact on the possibility to retrieve DNA profiles. As a matrix, it is complex and extremely heterogeneous. It consists of a variety of inorganic and organic compounds in different proportions depending on the soil type. The content of the soil affects the DNA extraction efficiency and subsequent extract quality [29]. For example, DNA can bind to soil particles where factors such as soil texture, cation concentration and pH influence the affinity [30]. Organic compounds that are not sufficiently removed during DNA extraction may disturb downstream processes such as PCR-based analysis. Humic substances (humus) are brownish compounds from decomposed organic matter that constitute the main part of the organic material in soil [31]. Humic acids are known to have a severely inhibitory effect on PCR by affecting the DNA polymerase activity [[32], [33], [34]]. Additionally, humic acids can act as a fluorescence inhibitor in qPCR, thereby affecting the sensitivity and accuracy of the analysis [34].

Most DNA extraction protocols for crime scene stains perform poorly even for minute amounts of soil [29,35]. Previously, efforts have been made to optimise DNA extraction from human cells present in 0.1-1 g of soil [[35], [36], [37]]. However, the spots indicated by search dogs are generally too large to ensure that any body fluid present will be captured in a soil sample of that size. The sampling is further complicated by the fact that the traces are often not visible to the naked eye. Thus, DNA extraction protocols that can handle larger amounts of soil and other environmental matrices are highly warranted. In addition, these protocols must enable efficient DNA extraction of various human cell types.

Here we present the optimisation, evaluation and implementation of a DNA extraction protocol for human body fluids in environmental matrices such as soil. The aim was to enable the extraction of human DNA in the presence of around 10 g of soil or corresponding volumes of other matrices. The protocol is intended for use in the investigation of crimes committed outdoors, in which crime scene search dogs are used to locate the stains. However, the protocol may be applied on any outdoor sample where human body fluids are expected. The protocol is based on the DNeasy PowerMax Soil kit (Qiagen) primarily developed for DNA extraction of microorganisms in soil [29,38]. For efficient lysis of sperm cells, the protocol was optimised by combining enzymatic, chemical and physical lysis strategies. The protocol was evaluated for semen, blood, saliva and cell-free DNA as well as for six different types of environmental matrices, from forest soil to gravel. Finally, the performance of the protocol in casework was investigated.

## 2 Materials and methods

This study was approved by the Swedish Ethical Review Authority (approval no 2024-01170-01).

### 2.1 Human cell samples

Volunteer donors provided samples of saliva, blood and semen under written informed consent. For each body fluid type, samples were obtained from three different volunteers in order to address variation between individuals. DNA extraction was performed in triplicates using 5 μL of each body fluid sample. For saliva and blood, female DNA was extracted using an in-house direct lysis protocol [39]. For semen an optimised protocol based on a procedure described in a user manual was applied [40]. In the latter protocol, dithiothreitol (DTT, D9779, Merck Sigma-Aldrich, Darmstadt, Germany) and Proteinase K (P2308, Merck Sigma-Aldrich) was added to a final concentration of 40 mM and 0.4 mg/mL, respectively. The DNA extracts were further purified using Amicon Ultra-2 30K (Merck Millipore, Darmstadt, Germany) and TE buffer (10 mM Tris, 0.1 mM EDTA, pH 8.0, Medicago AB, Uppsala, Sweden). All DNA extracts were quantified using the PowerQuant System (Promega Corporation, Madison, WI, USA) on a 7500 real-time PCR System (Thermo Fisher Scientific, Waltham, MA, USA). The persons providing the samples with median DNA yields for saliva (0.28 ± 0.020 ng/μL), blood (0.59 ± 0.018 ng/μL) and semen (4.6 ± 0.35 ng/μL), were chosen for further experiments.

### 2.2 Outdoor samples

Six types of outdoor samples were used as matrices: 1) *forest soil* from a mixed forest (broadleaved trees, spruce and pine, coordinates 58.56768, 15.28893, pH_H2O_ 5.12), 2) *sandy soil* from a pine forest (coordinates 55.58357, 13.08953, pH_H2O_ 8.33), 3) *park soil* from an urban park area (coordinates 58.40694, 15.62230, pH_H2O_ 6.22), 4) *sand* from the beach of a freshwater lake (coordinates 58.55156, 15.00498, pH_H2O_ 6.37), 5) *gravel* sized approximately 1-5 mm in diameter, collected from a car parking lot (coordinates 58.55729, 15.27928, pH_H2O_ 6.91) and 6) *plant material*, consisting of dry autumn leaves from birch and oak, collected from a mixed forest (coordinates 58.54034, 15.30755, pH_H2O_ 5.71). All environmental matrices were allowed to dry in room temperature before proceeding with the experiments. After drying, the leaves in the plant material were crumbled into smaller pieces.

### 2.3 Optimisation of the soil DNA extraction protocol

**Fig. 1:**
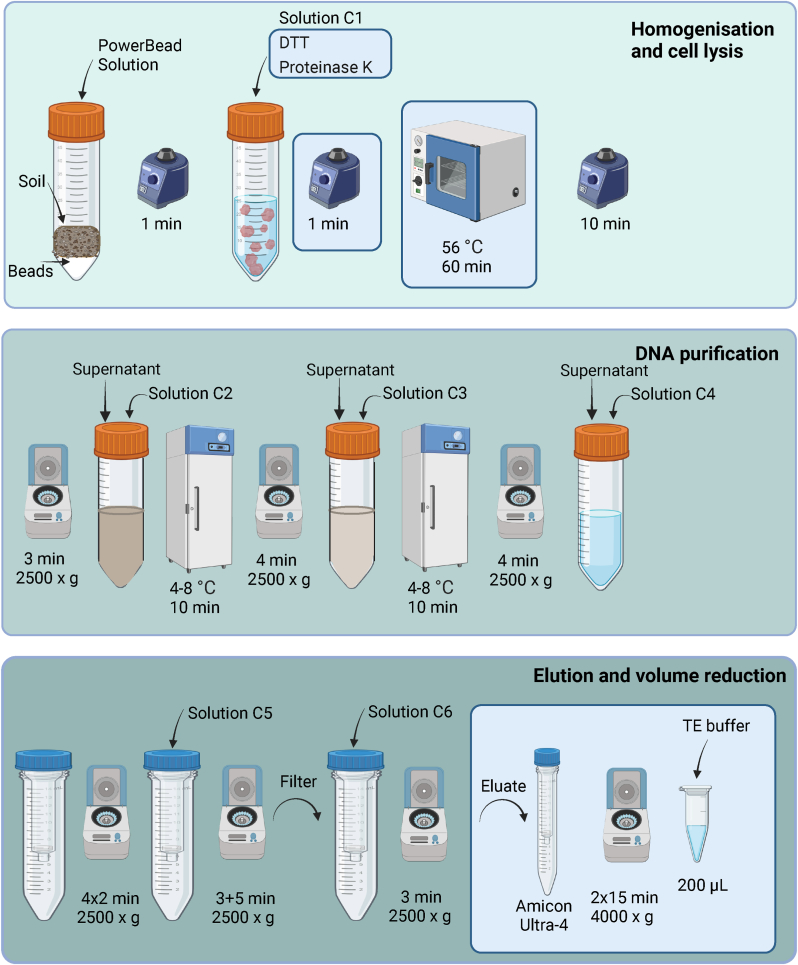
Illustration of the optimised soil protocol based on the DNeasy PowerMax Soil kit (Qiagen). Modifications to the original protocol are shown in blue boxes. First, the soil sample is homogenised and the cells lysed (top figure). Cell lysis is accomplished using a combination of chemical (chaotropic agent, anionic detergent and DTT), physical (mechanical bead beating and heat) and enzymatic (Proteinase K) treatments. DNA purification follows in several steps using different buffers where non-DNA material, e.g. proteins, forms a precipitate which is pelleted during centrifugation (middle figure). DNA left in the liquid phase is transferred to a new tube for further purification. The final part of the protocol is elution of DNA and volume reduction (bottom figure). The DNA is first bound to a silica membrane using a solution with high salt concentration (C4). The membrane is then washed with ethanol (C5) and the DNA eluted with 10 mM Tris buffer (C6). In the final step the eluate is further concentrated to 200 μL using a filter device. Created with BioRender.com.

Four modifications to the cell lysis step of the DNeasy PowerMax Soil kit (Qiagen, Hilden, Germany) were evaluated to optimise the DNA extraction of sperm cells (triplicates of 5 μL semen). In all experiments, the DNeasy PowerMax Soil kit extraction was performed according to the manufacturer's recommendations with the exception that the tubes were vortexed for 1 min instead of 30 s after the addition of solution C1 [38]. The tested modifications were: 1) addition of Proteinase K to a final concentration of 0.4 mg/mL, 2) addition of DTT to a final concentration of 40 mM, 3) addition of Proteinase K and DTT to final concentrations of 0.4 mg/mL and 40 mM, respectively, and 4) addition of Proteinase K and DTT to final concentrations of 0.4 mg/mL and 40 mM, respectively with an additional incubation step of 1 h at 56 °C (Fig. 1). A volume reduction step using AmiconUltra-4 30K (Merck Millipore) was integrated for all protocol variants to reduce the final volume from 5 mL to 200 μL.

### 2.4 DNA extraction of outdoor samples

The chosen optimised soil protocol based on the DNeasy PowerMax Soil kit (Fig. 1) with the addition of Proteinase K and DTT to final concentrations of 0.4 mg/mL and 40 mM, respectively with an additional incubation step of 1 h at 56 °C was further evaluated. DNA was extracted from semen in the presence of the different environmental matrices described in section 2.2. In addition, different amounts of *forest soil* were examined. The performance of the protocol was also evaluated for saliva, blood and cell-free DNA.

The environmental matrices were first transferred to tubes containing Power Beads. For *forest soil*, 5, 10, 15, 20 and 25 g of soil were tested in triplicates with 5 μL semen. For *sandy soil*, *park soil*, *sand*, *gravel* and *plant material*, 10 mL of the matrices were applied in triplicates with 5 μL semen. Additionally, triplicates of 5 μL saliva or blood were transferred onto 10 mL samples of *forest soil*. 300 μL of 2800M Control DNA (10 ng/μL, Promega) was added in triplicates with and without 10 mL of *forest soil* moistened with Milli-Q water (Merck Milli-Q, Darmstadt, Germany). After transferring body fluid or cell-free DNA onto the matrices the samples were let to dry overnight in room temperature with the lid off. All samples except the ones with 2800M were thereafter stored at −20 °C until DNA extraction.

The optimised soil protocol used for DNA extraction of human cells mixed with environmental matrices was as follows (Fig. 1). Add 15 mL Power Bead Solution (including the chaotropic agent guanidinium thiocyanate) to the tube containing the sample and Power Beads. The ceramic beads are chemically inert and will therefore not bind DNA during the bead beating process. Vortex the tube vigorously for 1 min. Check solution C1 (containing the anionic detergent SDS) for precipitation (if so, heat the bottle to 56 °C until dissolved). Add 1.2 mL solution C1, 600 μL DTT (1 M), 600 μL Proteinase K (10 mg/mL). Vortex the tube vigorously for 1 min. Incubate 60 min in a heating cabinet at 56 °C. Place the tube on the sample tube holder on the vortex and vortex at full speed for 10 min. These initial steps release the cells from the soil and the cells lyse. Nucleases are inactivated. During the following steps the DNA is purified. Centrifuge the tube at 2500×*g* for 3 min then transfer the supernatant to a new tube containing 5 mL solution C2. Turn the tube upside down twice to mix the contents. Incubate for 10 min in a refrigerator (4-8 °C). Centrifuge the tube at 2500×*g* for 4 min and then transfer the supernatant to a new tube containing 4 mL solution C3. Turn the tube upside down twice to mix the contents. Incubate for 10 min in a refrigerator (4-8 °C). Both C2 and C3 contain proprietary reagents that precipitate non-DNA molecules and thus remove PCR inhibitors. Centrifuge at 2500×*g* for 4 min and then transfer the supernatant to a new tube containing 30 mL of the high concentrations salt solution C4, allowing the DNA to bind to the filter. The C4 solution needs to be shaken prior to use. Turn the tube upside down twice to mix the contents. Transfer the lysate stepwise to the filter tube (part of the kit) and centrifuge at 2500×*g* for 2 min between the transfers. Discard the flow through and repeat until the entire volume has been processed. To avoid leakage during centrifugation, do not fill the tube to the rim. Add 10 mL of the ethanol-based wash solution C5 to the spin filter and centrifuge at 2500×*g* for 3 min. Discard the flow through. Centrifuge the spin filter at 2500×*g* for 5 min and carefully transfer the spin filter to a new tube. Add 5 mL of the sterile elution buffer C6 with 10 mM Tris and centrifuge at 2500×*g* for 3 min to release the DNA from the filter. Discard the spin filter and transfer the extract stepwise to an AmiconUltra-4 device (maximum 3.5 mL) for further volume reduction. Centrifuge at 4000×*g* for 5-15 min between each transfer. The time needed depends on the flow-rate of the liquid through the filter. Empty the collection device after each centrifugation. Transfer the eluate to a microfuge tube and add 1 x TE-buffer to a final volume of 200 μL.

### 2.5 DNA analysis

All DNA extracts were quantified using PowerQuant on a 7500 real-time PCR System, with standard curves of 0.001-50 ng/μL (PowerQuant Male gDNA Standard, 50 ng/μL) and a limit of quantification of 0.001 ng/μL. The short autosomal fragment of 84 bp included in PowerQuant was used to quantify the total amount of human DNA. When combined with the long autosomal fragment (294 bp) DNA degradation could be estimated where a ratio of [short fragment]/[long fragment] above 2 indicated DNA degradation. Internal PCR control (IPC) results were used to estimate PCR inhibition. An IPC Cq value larger than the mean IPC Cq value of the standard curve reactions plus 3 standard deviations was considered to indicate inhibition.

STR profiling was performed using the PowerPlex Fusion 6C System (Promega), Veriti Thermal Cycler, 3500xL Genetic Analyzer and GeneMapper ID-X v.1.6 software (Thermo Fisher Scientific) according to the manufacturer's recommendations or using accredited in-house protocols. Samples with DNA concentrations above 0.1 ng/μL were diluted prior to amplification. The electropherograms were evaluated based on amplification efficiency by calculating the total sum of allele peak heights (TPH) in relative fluorescence units (rfu) from all alleles present. For diluted samples, TPH were adjusted with the dilution factor. The quality of the electropherograms was evaluated by counting the number of successfully typed loci. In addition, the allele peak height of a long STR marker (Penta E) was divided by that of a short marker (D3S1583) both labelled with the same fluorescent dye. This ratio (TPH Penta E/TPH D3S1583) was used as an additional measure of PCR inhibition.

Strandedness and fragmentation of the extracted DNA from the different environmental matrices were investigated using the protocols presented by Jansson et al. [41]. A Qubit 2.0 Fluorometer with the Qubit ssDNA Assay Kit (Q10212) and the Qubit 1X dsDNA HS Assay Kit (Q33230, Thermo Fisher Scientific) were used to measure DNA strandedness. In addition, the total amount of DNA (human and non-human) was measured using the dsDNA assay. Prior to analysing ssDNA, dsDNase (EN0771, Thermo Fisher Scientific) was used to degrade any remaining dsDNA by incubating 2 μL of each sample at 37 °C for 5 min. DNA fragmentation was investigated by using the 5200 Fragment Analyzer system with HS Genomic DNA 50 kb kit (DNF-468-500, Agilent Technologies, Santa Clara, CA, United States). Fragmentation was evaluated by analysing the average fragment size of the highest peak as well as Genomic Quality Number (GQN) with a threshold of 10 kb. GQN ranges from 1 to 10, and indicates the amount of fragments with fragment sizes above the set threshold value. As an example, a GQN of 5 indicates that 50% of the fragments are above 10 kb.

Massively parallel sequencing (MPS) was performed using the ForenSeq Imagen Kit with the Enhanced PCR1 reaction mix and the MiSeq FGx System (Qiagen) according to the manufacturer's recommendations. Analysis was performed for samples including only 5 μL semen, as well as samples with semen and 5 g or 10 g of *forest soil* (n = 2). In addition, the DNA preparations from DNA extraction of 10 g *forest soil* were reanalysed with the following adjustments; reduced template input (from 1 ng to 0.6 ng DNA), doubling the amount of enzyme mix in the PCR reaction (from 0.3 to 0.6 μL) and purification of the DNA extract using an in-house phenol-chloroform protocol. The quality of the sequencing results using ForenSeq Imagen Kit was evaluated by calculating the total number of reads and the number of successfully typed single nucleotide polymorphism (SNP) markers.

### 2.6 Outcome in casework

After implementation at the Swedish National Forensic Centre, the optimised soil DNA extraction protocol for environmental matrices has been used in 34 cases consisting of 11 rapes, 18 murders and 5 other serious crimes (attempted murder, aggravated assault, arson and kidnapping). From these 34 cases, 51 items were recovered, giving a mean of 1.5 items per case. These included environmental samples (soil, sand, gravel and plant material), items found outdoors e.g. clothes with visible dirt and partly decomposed fruits or berries recovered indoors. In total 81 samples have been extracted using the optimised soil protocol, giving a mean of 1.6 samples per item. In at least 26 of the 34 cases crime scene dogs were used in the search for human biological traces.

### 2.7 Data analysis

DNA results are presented as mean values ± standard deviation. Due to the large differences in observed DNA yield between protocols, a logarithmic transformation was applied to the data to normalize variances. Initial data analysis indicated no obvious deviations from (approximately) normally distributed data and equal variances, checked by Q-Q plots and Levene's test in R package car (96% of points within 95% confidence envelopes and p-values >0.4 respectively) [42]. Differences between groups were tested statistically with one-way ANOVA (significance level 0.05) followed by independent two-sample post-hoc t-tests assuming equal variances and using Bonferroni correction. Six t-tests were performed for the optimisation experiment (section 3.1) and four t-tests for the evaluation of different quantities of soil (section 3.2), giving significance levels of 0.0083 (0.05/6) and 0.013 (0.05/4), respectively.

## 3 Results

### 3.1 Optimisation of the soil DNA extraction protocol for sperm cell lysis

**Fig. 2:**
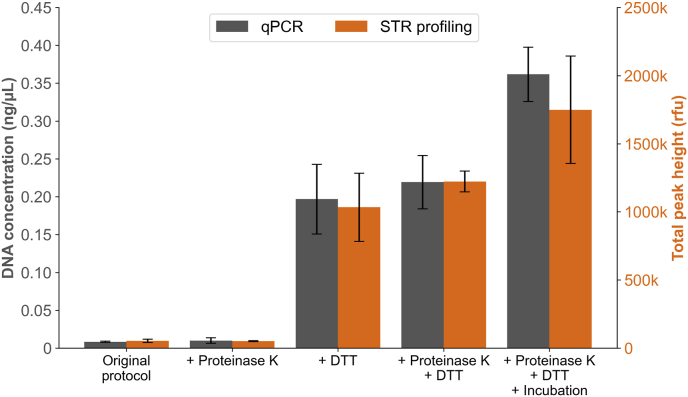
Mean DNA concentrations (ng/μL) and mean total peak heights (rfu) for 5 μL semen extracted using different modifications of the soil kit. The original protocol was compared to the modified protocols including the addition of Proteinase K, DTT and a heat incubation step. All protocols including DTT gave significantly higher DNA yields compared to the original protocol (only DTT: p = 3 × 10^−5^, DTT and Proteinase K: p = 8 × 10^−6^, DTT, Proteinase K and incubation: p = 1 × 10^−6^). Error bars show standard deviation, n = 3.

The original DNeasy PowerMax Soil protocol was optimised for DNA extraction of human crime scene stains, in particular sperm cells. The modifications consisted of adding Proteinase K and the reducing agent DTT alone or in combination, as well as an additional heat incubation step (Fig. 1). The ANOVA test indicated significant differences in yields between protocols (p = 1.4 × 10^−9^). The combination of all three modifications gave the highest mean DNA concentration when compared to the original protocol, increasing the yield 43-fold (Fig. 2). The addition of both DTT and Proteinase K, but without the extra incubation step, gave somewhat lower mean DNA yield compared to the combination of all three modifications. However, the difference was not statistically significant when using the Bonferroni correction (p = 0.011). Looking specifically at the effect of Proteinase K, slightly higher mean concentrations were generated when this was included in the protocols. However, the differences were not statistically significant (mean DNA concentration 0.008 ± 0.001 ng/μL for the original protocol vs. 0.010 ± 0.003 ng/μL when adding only Proteinase K (p = 0.59), and 0.20 ± 0.038 ng/μL when adding DTT vs. 0.22 ± 0.029 ng/μL when adding Proteinase K and DTT (p = 0.53)). Complete and well-balanced STR profiles were generated for all different modifications, with DTT showing the strongest effect on DNA yield. The total peak heights of all STR alleles correlated well to the DNA concentrations (Fig. 2). There was no indication of PCR inhibition or DNA degradation when studying the PowerQuant results. The protocol including all three modifications (i.e. Proteinase K, DTT and heat incubation) was selected for subsequent experiments. This protocol is referred to as the optimised soil protocol.

### 3.2 Maximum quantity of soil in the DNA extraction

**Fig. 3:**
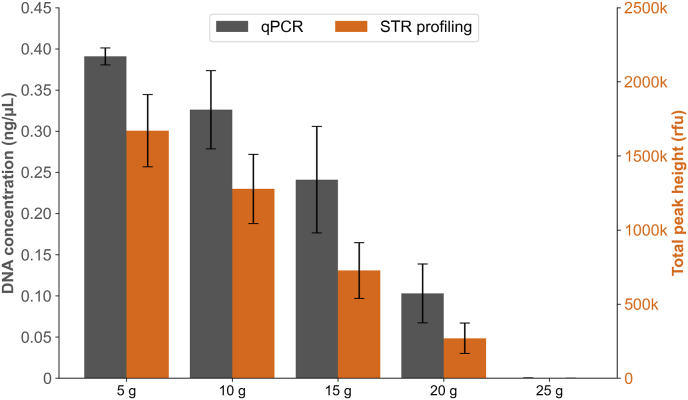
Mean DNA concentrations (ng/μL) and mean total peak heights (rfu) for 5 μL semen with different quantities of *forest soil* extracted using the optimised soil protocol. Error bars show standard deviation, n = 3.

Different quantities of *forest soil* with 5 μL semen were extracted to evaluate the maximum input of environmental matrix for the optimised soil protocol. Fig. 3 shows lowered mean DNA concentrations and mean total peak heights with increasing quantities of *forest soil*. The ANOVA test indicated significant differences in yields between quantities (p = 4.5 × 10^−4^). For 10 g and 15 g soil the yields were somewhat lower compared to 5 g, but these differences were not statistically significant (p = 0.097 and p = 0.038, respectively). However, increasing the quantities to 20 g and 25 g soil gave significantly lowered yields (p = 0.002 and p = 5 × 10^−8^ respectively). Complete STR profiles were generated for up to 20 g of *forest soil*, although individual replicates showed signs of PCR inhibition when 15 g was added. The inhibition effect was demonstrated by lower peak heights for the longer STR markers and IPC Cq values slightly over the threshold for inhibition. When adding 25 g of soil only single STR alleles (3, 1 and 0 alleles per replicate) with low peak heights (<200 rfu) were detected. During DNA extraction of 25 g soil, most of the buffer was absorbed and therefore less volume of supernatant was available for transfer to the next step. In addition, there was a visible brownish colour gradient in the DNA extracts, increasing in intensity as more *forest soil* was added, showing that the removal of humic substances was incomplete.

A balance may not be available at a routine DNA trace recovery laboratory. It may therefore be handier to add a certain volume of matrix to the tube instead of a certain weight. In addition, this would simplify the trace recovery of materials with different densities and ensure that enough lysis buffer is available also for light matrices such as dry leaves. Here, 13 g of dry *forest soil* corresponded to 10 mL and fell within the range where successful results were generated. Therefore 10 mL of environmental material was further used in this study.

### 3.3 Different types of outdoor samples

Samples collected from outdoor crime scenes can vary greatly in content of organic matter and particle size. Six different matrices representing different environments (*forest soil*, *sandy soil, park soil, sand, gravel and plant material*) were therefore selected and evaluated. When 10 mL of each environmental matrix was extracted in combination with 5 μL semen using the optimised soil protocol, all samples gave well-balanced STR profiles with all expected alleles detected. The ratios comparing allele peak heights of a long marker with a short marker labelled with the same fluorescent dye were 69% or higher. PowerQuant did not indicate any PCR inhibition or DNA degradation (Table 1).

**Table 1:** DNA extraction of semen with different types of outdoor samples using the optimised soil protocol. Results from PowerQuant are presented as the mean human DNA concentration, the internal PCR control (Cq values) and degradation index. From STR typing the numbers of successfully typed loci (maximum 27) are shown. In addition, the mean total peak height in relative fluorescence units (rfu) and the ratio when dividing a long STR marker (Penta E) with a short marker (D3S1583) labelled with the same fluorescent dye are presented. From Qubit measurements, the mean total DNA concentration (ng/μL) and DNA strandedness given as the percentage of dsDNA in the DNA extracts are shown. DNA fragmentation is assessed by the average fragment size of the major DNA peak using Fragment Analyzer. Genomic quality number (GQN) from Fragment Analyzer using a threshold of 10 kb is also presented. Semen without any environmental sample is used as reference. Mean values ± standard deviations are presented, n = 3.

Environmental matrix type	Human DNA concentration (ng/μL)	Internal qPCR control (Cq)^a^	Degradation index^b^	Number of successfully typed loci	Total peak height (krfu)^c^	Penta E/D3S1583	Total dsDNA concentration (ng/μL)	dsDNA (%)	Fragment size (kb)	GQN
Reference – only semen	0.36 ± 0.029	20.37-20.61	1.1 ± 0.1	27 ± 0	1700 ± 320	1.1 ± 0.1	0.37 ± 0.043	>95	42 ± 6.6	6.7 ± 0.36
Forest soil (pH_H2O_ 5.12)	0.33 ± 0.039	20.96-21.04	1.4 ± 0.2	27 ± 0	1300 ± 190	0.7 ± 0.1	190 ± 17	>95	6.8 ± 1.0	1.8 ± 0.45
Sandy soil (pH_H2O_ 8.33)	0.85 ± 0.10	20.41-20.47	1.0 ± 0.1	27 ± 0	5500 ± 630	0.9 ± 0.1	170 ± 25	>95	5.0 ± 1.2	1.4 ± 0.62
Park soil (pH_H2O_ 6.22)	0.38 ± 0.086	20.38-20.69	1.1 ± 0.1	27 ± 0	2100 ± 430	0.9 ± 0.1	52 ± 2.9	>95	7.6 ± 1.7	2.7 ± 1.0
Sand (pH_H2O_ 6.37)	0.41 ± 0.074	20.43-20.57	1.0 ± 0.0	27 ± 0	2600 ± 1100	1.0 ± 0.0	6.0 ± 0.32	>95	29 ± 6.7	5.5 ± 0.59
Gravel (pH_H2O_ 6.91)	0.78 ± 0.11	20.49-20.71	1.0 ± 0.0	27 ± 0	5300 ± 240	0.8 ± 0.0	19.5 ± 2.5	>95	7.0 ± 0.70	2.1 ± 0.46
Plant material (pH_H2O_ 5.71)	0.53 ± 0.12	20.35-20.40	1.1 ± 0.1	27 ± 0	3000 ± 210	0.8 ± 0.1	14.6 ± 8.1	>95	7.6 ± 0.98	2.1 ± 0.42

a Cq > 21.06 indicates PCR inhibition.

b Degradation index >2 indicates DNA degradation.

c For samples that were diluted prior to PCR, the total peak height has been adjusted with the dilution factor.

The DNA yields varied between the outdoor sample types, presented as human DNA qPCR results (PowerQuant) and as the total peak heights of the STR profiles. The analysis of total DNA content using Qubit demonstrated a high proportion of non-human DNA (Table 1). In particular, the different soil samples showed up to 190 ng/μL total DNA, meaning that up to 99.8% of the DNA present originated from microorganisms with possible minor contributions from plants and animals. The human DNA concentration was equivalent to the total DNA concentration for the samples including only semen, without any environmental sample added.

When analysing the samples using the MPS-based ForenSeq Imagen Kit, all 107 SNP markers were successfully typed for the samples with only semen. The total numbers of reads were above 100 k. Analysing DNA extracts from semen in the presence of *forest soil*, resulted in zero reads. This was regardless of any adjustments made to lower the amount of input template, increase the amount of DNA polymerase or purify the DNA extracts using phenol-chloroform.

Studying the overall quality of the DNA extracts, the average fragment sizes of the major DNA peaks were between 5.0 and 7.6 kb for the different matrices. Less than 30% of the fragments were above 10 kb (GQN <3) for all matrices, except for *sand*. *Sand* showed a mean fragment size of 29 kb and a GQN of 5.5. The reference sample, containing only semen, showed larger fragment sizes (42 kb). Since most of the DNA from the outdoor samples is microbial, the quality estimates for these represent microbial DNA rather than human DNA. More than 95% of the DNA was double stranded (Table 1).

### 3.4 Different types of human samples

Saliva or blood (5 μL), was extracted with 10 mL of *forest soil* giving mean DNA yields of 0.031 ± 0.005 ng/μL for saliva and 0.15 ± 0.036 ng/μL for blood. Two of the three replicates for both saliva and blood indicated partial PCR inhibition using PowerQuant. One replicate for saliva had a degradation index above 2, which is likely an effect of inhibition as indicated by the IPC Cq value. All expected alleles in all STR profiles were detected. However, for saliva some of the longer markers, e.g. Penta E, had lower peak heights compared to the shorter ones, also indicating slight PCR inhibition (Table 2).

**Table 2:** DNA extraction of saliva and blood using the optimised soil protocol. Results from PowerQuant are presented as the mean human DNA concentration, the internal PCR control Cq values and degradation index. For STR typing the numbers of successfully typed loci (maximum 24 for females) are shown. In addition, the mean total peak height in relative fluorescence units (rfu) and the ratio when dividing a long STR marker (Penta E) with a short marker (D3S1583) labelled with the same fluorescent dye are presented. Mean values ± standard deviations are presented, n = 3.

Human body fluid	Human DNA concentration (ng/μL)	Internal qPCR control (Cq)^a^	Degradation index^b^	Number of successfully typed loci^c^	Total peak height (krfu)^d^	Penta E/D3S1583
Saliva	0.031 ± 0.005	20.47-20.96	1.8 ± 0.36	24 ± 0	130 ± 14	0.08 ± 0.03
Blood	0.145 ± 0.036	20.58-21.00	1.3 ± 0.22	24 ± 0	490 ± 27	0.80 ± 0.16

a Cq > 20.84 indicates PCR inhibition.

b Degradation index >2 indicates DNA degradation.

c Saliva and blood from females.

d For samples that were diluted prior to PCR, the total peak height has been adjusted with the dilution factor.

The ability of the optimised soil protocol to extract cell-free DNA was investigated by adding approximately 2 μg of reference DNA (2800M) to tubes with and without 10 mL of *forest soil*. The mean DNA yield for the extracts without soil was 2.1 ± 0.46 ng/μL and with soil 0.41 ± 0.082 ng/μL. This gives 20% recovery with only cell-free DNA and 4% with 10 mL *forest soil*. PowerQuant showed inhibition for the samples including soil, with IPC Cq value shifts of 0.30-0.66 in relation to the threshold for inhibition.

### 3.5 Analysis of outdoor samples in routine casework

**Fig. 4:**
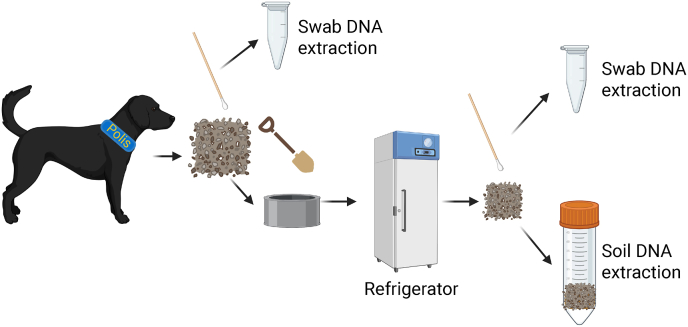
The recommended procedure for recovering outdoor traces located by a crime scene dog and preparing them for DNA extraction. Trace recovery is preferably performed at the outdoor crime scene with a dual approach, using a swab which proceeds to presumptive testing and DNA extraction. A soil sample is thereafter recovered. Ideally, the soil sample is punched out from the ground using e.g. a plastic container with a lid. This prevents any mixing during transportation. This sample is kept in a refrigerator. At the laboratory, trace recovery may then be performed using a swab which proceeds to presumptive testing and DNA extraction. If swabbing is not possible, or expected to give suboptimal results, it is advised to take up to 10 mL of the environmental matrix for extraction using the optimised soil protocol. Created with BioRender.com.

A procedure for recovering outdoor traces located by crime scene dogs and preparing them for DNA extraction has been established (Fig. 4). If possible, we recommend to initially swab the area marked by the crime scene dog before punching an environmental sample using a plastic container, as described by Johnzon [27]. Both the swab and the outdoor sample would then be transported to the laboratory for further processing. The outdoor sample may either be swabbed again or a sample of up to 10 mL may be taken for soil DNA extraction using the optimised soil protocol.

In casework, a total of 81 samples from 51 items recovered in 34 cases of serious crimes have so far been processed using the optimised soil protocol. DNA was retrieved from 23 of 51 items (45%). Of these, 20 items gave usable STR profiles according to internal guidelines. The remaining three gave results too complex for further evaluation, mainly because of too many contributors in combination with low peak heights (Table 3). Usable STR profiles were generated in 50% of the cases (17 of 34), of which 82% only gave STR profiles when using the optimised soil protocol. For the remaining 18%, initial swabbing of the samples also gave usable STR profiles. Swabbing gave DNA results in one case where the optimised soil protocol failed. In one case the STR profile matched the suspect. Six STR profiles matched the victim or other persons connected to the case. In total, 8 STR profiles have been added to the national crime scene sample DNA database, with no hits at the time of writing.

**Table 3:** Application of the optimised soil protocol in casework. The optimised soil protocol has been used in different cases including rape (R), murder (M) and other serious crimes (O) (attempted murder, arson, kidnapping and aggravated assault). Results are presented as the number of items from which DNA was successfully retrieved, either as usable STR profiles or complex mixtures (too many contributors in combination with low peak heights). The usefulness in criminal cases is presented as the number of trace profiles added to the national crime scene sample DNA database and the number of matches within the cases. This is presented either as a match to a suspect or to another person connected to the case, e.g. a victim.

Type of matrix	Number of cases & type of case (R/M/O)	Number of items	DNA retrieved	Usable STR profile	Complex mixture	STR profile added to the database	Match to a suspect within the case	Match to a non-suspect within the case (e.g. victim)
Soil/sand/gravel	4 R, 9 M, 1 O	23	6	6	-	2	-	4
Plant material	7 R, 5 M, 2 O	20	14	12	2	6^b^	1	-
Item collected outdoor	0 R, 3 M, 2 O	5	1	-	1	-	-	-
Fruit or berries^a^	0 R, 1 M, 0 O	3	2	2	-	-	-	2
Total	11 R, 18 M, 5 O	51	23	20	3	8	1	6

a One case where partly decomposed fruit and berries were recovered indoors. Other in-house DNA extraction methods did not generate any usable STR profiles and therefore the optimised soil protocol was applied.

b For some of the usable STR profiles retrieved, the same profile was also found on other items in the case using different protocols.

## 4 Discussion

This study demonstrates the successful adaptation of a protocol originally developed for microbial DNA extraction to the forensic context of human identification. The optimised soil protocol has been used in the investigation of serious crimes committed outdoors, where crime scene dogs have been used to search for human body fluids. STR profiles were generated from crime scene samples in 50% of the cases. In the majority of these (82%), no results were generated using swabs and traditional DNA extraction methods. For the remaining 50% of cases, it remains unknown whether the samples contained any amplifiable human DNA or not, despite being positively indicated by the dogs. As shown in earlier studies, dogs are capable of detecting body fluids deposited outdoors, even when neither DNA profiling nor presumptive testing yields positive results [26,43]. One explanation may be that the odour persists longer than the DNA molecules. In addition, the fact that some DNA is inevitably lost during sampling and DNA extraction may contribute to this outcome. The full range of factors and mechanisms involved in the canine olfactory system is not yet fully understood [44]. Olfactory detection begins in the olfactory neuroepithelium, located within the nasal cavity. Odorant molecules bind to olfactory receptors regulated by a large family of olfactory receptor genes [45]. The interaction triggers an electrical signal that is transmitted to the olfactory bulb in the brain. This results in the formation of a unique neural representation, or “odour signature”, of the detected substance [46]. Considering the nature of DNA and the fact that it is situated within the cell nuclei in intact cells, it is unlikely that the DNA itself contributes to the odour signature. Thus, even with optimal sampling and DNA extraction methods, some traces pointed out by crime scene dogs will not provide any amplifiable human DNA, e.g. due to degradation.

Skalleberg et al. described a procedure of using crime scene dogs, presumptive testing and swabbing in the field [26]. Our process builds on their work by offering the possibility of performing swabbing also in the laboratory through punching of a part of the ground. Also, DNA extraction may be executed using the soil if swabbing is not feasible, or gives no usable results. The punching procedure using a plastic container was developed by a crime scene investigator (CSI) in 2013 [27]. This procedure has since been part of the CSI training in Sweden. The optimised soil protocol enabled DNA extraction of various human cell types from around 10 mL of different environmental matrices. The matrices spanned from pH 5.12 to 8.33 and consisted of organic material (*plant material*), both organic and inorganic material (*soil*) and inorganic material (*sand* and *gravel*). The matrices had different textures, with different total surface areas. For example, the larger gravel grains have a lower total surface area for a given volume, compared to the minute grains of sand. All these characteristics may influence DNA binding and extraction efficiency, and thus also the DNA yield. The results indicate a higher DNA yield for *gravel* compared to sand (0.78 ng/μL vs. 0.41 ng/μL). This correlates with the idea that a larger surface area provides more binding sites for the DNA, as discussed by Levy-Booth et al. [30]. Additionally, the *sandy soil* gave higher yields compared to the other soil types, possibly by providing fewer binding sites. On the contrary, the *sandy soil* gave equivalent DNA yield (0.85 ng/μL) compared to *gravel* and higher yield than *sand*.

All matrices in the study exhibited pH values above 5, which is the isoelectric point of DNA [30,47]. At lower pH, the phosphate groups in the molecule's backbone are partly protonated, lowering its net negative charge. This makes binding of DNA to the negatively charged surfaces of clay minerals more likely, although still necessitating divalent ions such as Mg^2+^ or Ca^2+^ to form cationic bridges. Therefore, the binding affinity of free DNA to soil particles is influenced by both the pH and the availability of divalent cations up to a certain pH [29]. We observe a correlation between increased yield of human DNA with increased pH for soils and inorganic matrices. This is in line with the results from a previous study where pH of the lysis buffer was evaluated (pH 6-10). The DNA yield from soil decreased with decreasing pH of the lysis buffer within this range. However, pH 9 was recommended over pH 10 due to the release of larger amounts of humic substances at pH 10 [48]. Even though this is equivalent to the pH of the lysis buffer included in DNeasy PowerMax Soil kit (pH 9) [49], the pH of the soil could be a contributing factor of the DNA yield observed in the results.

The strong absorption of cell-free DNA to soil was evident in this study. When DNA added to moistened *forest soil* was left over night, there was an 80% decrease in DNA yield compared to when no soil was present. To prevent cell-free DNA from binding to the soil during DNA extraction, chelating agents such as ethylenediaminetetraacetic acid (EDTA) can be added. EDTA binds divalent cations and thereby prevents the formation of cation bridges [29]. It is common to add sodium phosphate to lysis buffers. This additive competes with the DNA molecules for binding sites. Another alternative is to pre-treat the soil with RNA [29,48]. Whether any of these components are included in the lysis buffer of the DNeasy PowerMax Soil kit is not known and likely considered a trade secret. The microbial DNA could, in our application, have the same effect, competing with the human DNA for binding sites. However, no positive correlation between human DNA yield and the presence of higher amounts of non-human DNA was observed in this study.

We conclude that the presence of large quantities of microbial DNA does not affect the efficiency of the optimised soil protocol in regard to human DNA yield. When the human DNA represented as little as 0.2% of the total amount of DNA, the yield was comparable to that obtained in the absence of environmental matrix. In addition, the downstream PCR process was not affected by large quantities of non-target DNA. The latter is in correlation with forensic Y-STR kits where male Y-STR profiles are successfully generated in the presence of large quantities of female DNA [50,51].

The amounts of humic substances in the samples are unknown, although their presence was visually confirmed by the colour of the DNA extracts. Complete STR profiles were generated for samples containing 5 to 20 g of *forest soil*. In the developmental validation of PowerPlex Fusion 6C, complete profiles were generated for extracts containing 100 ng/μL humic acid and partial profiles for 200 ng/μL and 300 ng/μL [52]. Using the ForenSeq Imagen Kit, DNA extracts from 5 g or 10 g soil gave no reads at all, despite efforts to optimise the PCR. As reported by Sidstedt et al. [53], commercial MPS kits are typically less inhibitor-tolerant than established capillary electrophoresis STR kits. ForenSeq Imagen Kit has been shown to provide good quality results for up to 50 ng/μL of humic acid when using the enhanced PCR1 buffer [54]. Based on the results for PowerPlex Fusion 6C and ForenSeq Imagen, the extracts from the 5 g and 10 g soil samples analysed in this study thus likely contained between 50 ng/μL and 200 ng/μL humic acid. This illustrates that the optimised soil protocol reduces, but does not remove, the need for a robust PCR chemistry. Further, the findings indicate a specific need to further improve the inhibitor-tolerance of forensic MPS-based typing methods.

Information on DNA quality is needed when considering using more sophisticated sequencing methods such as whole genome sequencing, e.g. since some library preparation require double-stranded DNA. As reported by Jansson et al. [41], the DNA extraction protocol can affect DNA fragmentation and DNA strandedness. Following processing with the optimised soil protocol, more than 95% of the DNA was double-stranded. This is in line with previous results for magnetic bead-based methods and DNA extraction using phenol-chloroform [41]. The fragment sizes and GQN values from samples including only semen, without environmental matrix, also correlated to the results for those methods.

DNA extraction protocols typically use one of two strategies to obtain a pure, high-yield DNA extract. The first approach involves the separation of intact cells from the matrix prior to cell lysis, using methods such as sedimentation or buoyant density centrifugation. This strategy has the advantage of removing cells before the DNA can adsorb to the matrix [55,56]. However, depending on their density, cells may become trapped within the soil or sediment. In contrast, the second approach begins with an initial lysis step to release DNA directly into the liquid phase, followed by sequential purification steps. This strategy is generally considered to be more efficient and is commonly employed by commercial kits for soil DNA extraction [29].

During DNA extraction, it is of course crucial to lyse the target cells. The cell membrane and the packaging of DNA in the cell nucleus varies among different human cell types. The nucleus of spermatozoa is embedded in the acrosome protected by the acrosomal cap. The DNA is bound to protamines connected by disulfide bonds [57,58]. These structures make them more resistant to lysis compared to cells in other human body fluids. It was obvious that the original protocol could not lyse the spermatozoa efficiently. This was improved by incorporating enzymatic, chemical and physical lysis strategies to the protocol, increasing the yield 43-fold.

## 5 Conclusions

By modifying a kit primarily developed for DNA extraction of microorganisms in soil, we were able to implement a protocol for human body fluids recovered outdoors. The protocol has been successfully used in forensic investigations of serious crimes. In addition, we present a process starting from a positive detection by a crime scene dog to a sample ready for DNA extraction. With this process it is possible to recover DNA from outdoor crime scenes in a time-efficient manner in order to limit the DNA degradation process. High quality STR profiles were generated from semen, blood and saliva in 10 mL of environmental matrix, such as soil, sand, gravel and dry plant material. However, the DNA extracts may still contain humic substances, possibly causing inhibition in MPS methods. The protocol yields dsDNA with equal quality as DNA extracted using magnetic bead-based methods or phenol-chloroform.

## CRediT authorship contribution statement

**Christina Forsberg:** Writing – original draft, Visualization, Validation, Methodology, Investigation, Formal analysis, Data curation, Conceptualization. **Ronny Hedell:** Formal analysis, Investigation, Writing – review & editing. **Ricky Ansell:** Writing – review & editing, Supervision, Investigation, Conceptualization. **Johannes Hedman:** Writing – review & editing, Supervision, Investigation, Conceptualization.

## Declaration of competing interest

The authors declare that they have no known competing financial interests or personal relationships that could have appeared to influence the work reported in this paper.
